# Effect of a palliative care program on trends in intensive care unit utilization and do-not-resuscitate orders during terminal hospitalizations. An interrupted time series analysis

**DOI:** 10.5935/0103-507X.20180042

**Published:** 2018

**Authors:** João Gabriel Rosa Ramos, Fernanda Correia Tourinho, Patrícia Borrione, Paula Azi, Tuanny Andrade, Vanessa Costa, Zan Reis, Paulo Benigno Pena Batista, Ana Verena Mendes

**Affiliations:** 1 Hospital São Rafael - Salvador (BA), Brasil.

**Keywords:** Palliative care, Resuscitation orders, Patient care planning, Interrupted time series analysis, Intensive care units

## Abstract

**Objective:**

To assess the effect of the implementation of a palliative care program on do-not-resuscitate orders and intensive care unit utilization during terminal hospitalizations.

**Methods:**

Data were retrospectively collected for all patients who died in a tertiary hospital in Brazil from May 2014 to September 2016. We analyzed the frequency of do-not-resuscitate orders and intensive care unit admissions among in-hospital deaths. Interrupted time series analyses were used to evaluate differences in trends of do-not-resuscitate orders and intensive care unit admissions before (17 months) and after (12 months) the implementation of a palliative care program.

**Results:**

We analyzed 48,372 hospital admissions and 1,071 in-hospital deaths. Deaths were preceded by do-not-resuscitate orders in 276 (25.8%) cases and admissions to the intensive care unit occurred in 814 (76%) cases. Do-not-resuscitate orders increased from 125 (20.4%) to 151 (33%) cases in the pre-implementation and post-implementation periods, respectively (p < 0.001). Intensive care unit admissions occurred in 469 (76.5%) and 345 (75.3%) cases in the pre-implementation and post-implementation periods, respectively (p = 0.654). Interrupted time series analyses confirmed a trend of increased do-not-resuscitate order registrations, from an increase of 0.5% per month pre-implementation to an increase of 2.9% per month post-implementation (p < 0.001), and demonstrated a trend of decreased intensive care unit utilization, from an increase of 0.6% per month pre-implementation to a decrease of -0.9% per month in the post-implementation period (p = 0.001).

**Conclusion:**

The implementation of a palliative care program was associated with a trend of increased registration of do-not-resuscitate orders and a trend of decreased intensive care unit utilization during terminal hospitalizations.

## INTRODUCTION

A large proportion of a population's deaths occurs in hospitals,^(1-4)^ and in Western countries, many in-hospital deaths are preceded by intensive care unit (ICU) admissions during hospitalization.^(3,5)^ Even in patients with severe, advanced diseases, ICU utilization during terminal hospitalization may occur in up to 50% of cases,^(6)^ and much of that care may be seen as nonbeneficial or inconsistent with patients' values and preferences.^(7)^ Moreover, potentially inappropriate ICU admissions^(8)^ may increase the strain on the allocation of scarce critical care resources.^(9-11)^

There is great variability in decisions of prognostication and the limitations of medical treatment,^(5,12)^ and it has been suggested that palliative care (PC) interventions can modify decisions about care goals and patient allocation.^(13-15)^ For instance, advanced care planning or PC referral in the wards or during an ICU stay may reduce inappropriate ICU admissions.^(14)^ It has been proposed that one of the main drivers of this change is timely and sensitive communication about appropriate goals of care, taking into consideration the patient's condition, prognosis and values.^(16)^

Even though recommended by international organizations, PC availability and utilization varies widely and may be especially infrequent in developing countries.^(17-19)^ However, there are few studies in developing countries describing ICU utilization during terminal hospitalizations and the impact of PC interventions in this population.^(20-25)^

In this study, we sought to evaluate the effect of the implementation of a PC program in trends of do-not-resuscitate (DNR) orders and ICU utilization during terminal hospitalizations. Moreover, we analyzed if this effect could be different in patients admitted to oncological or non-oncological specialties.

## METHODS

This study was approved, with a waiver for informed consent, by the Ethics Committee of *Hospital São Rafael* (HSR).

In-hospital deaths in the period from May 2014 to September 2016 (29 months) were included in the study. In the case of multiple hospital admissions in the study period, only the last admission was included.

*Hospital São Rafael* is a private, not-for-profit hospital with 350 beds in the northeast of Brazil. Intensive care units have an open admission policy, in which the referring physician determines ICU admission of the patient, except in moments of scarcity of available beds, when patients may be subjected to triage.

In April 2014, a flag in the electronic health record was created to identify patients with DNR orders; however, no standard policy or PC team existed. In September 2015, an institutional PC program was created and a program to increase institutional awareness was initiated. The main purposes of the palliative care program were to promote care for all dimensions of suffering, while respecting the autonomy of patients and relatives, and to better standardize goals of care, facilitating interdisciplinary communication and identification of end-of-life patients. Later, in April 2016, a PC physician, together with an intensivist, an oncologist, a pediatrician, a nurse, a social worker and psychologists began rounds on hospitalized patients as consultants, but not admitting patients as the primary team.

Data on demographic and clinical variables were collected retrospectively from electronic health records (MV Informatica Nordeste Ltda., Recife, Brazil). We also collected information on DNR order registrations and ICU admissions during the same hospitalization.

A DNR order registration was defined as the registration of a DNR order, as per the PC program in the electronic health record. ICU admission during the same hospitalization was defined as any admission to the ICU of a patient who died in the same hospitalization. Patients were defined as admitted to an oncological specialty if their primary admitting team was oncology, pediatric oncology or surgical oncology.

We evaluated the effect of the implementation of a PC program on the proportion of patients with in-hospital deaths that had a DNR order placed and the proportion of patients with in-hospital deaths that had been admitted to the ICU during hospitalization.

For that purpose, we analyzed the number and proportion of DNR registrations before and after implementation of the PC program. As a pre-specified secondary analysis, we also analyzed the proportion of DNR registrations stratified by oncological or non-oncological specialties. Moreover, we analyzed the number and proportion of ICU admissions during terminal hospitalization before and after implementation of the PC program.

### Statistical analysis

Categorical variables were described as proportions. Continuous variables were described as the median (interquartile range) or the mean ± standard deviation. Proportions were evaluated with chi-square statistics. Continuous variables were evaluated with Mann-Whitney U test or t-test.

To evaluate differences in DNR orders and ICU utilization over time, we performed chi-square tests for trend. To control for secular trends, we utilized interrupted time series analyses using autoregressive integrated moving average models, as previously described.^(26)^ Interrupted time series analysis is a quasi-experimental design that can evaluate the effect of an intervention using longitudinal data series.^(27)^ The presence of seasonal trends was evaluated visually and by examining the partial autocorrelation function of the model. For all analyses, the pre-implementation phase was defined as the 17 months before the implementation of the PC program (May 2014 to September 2015), and the post-implementation phase was defined as the 12 months after the implementation of the PC program (October 2015 to September 2016).

A two-tailed p value of less than 0.05 was considered significant in all analyses performed. Statistical analyses were performed with Statistical Package for Social Science (SPSS), version 21.0 (SPSS Inc., USA).

## RESULTS

From May 2014 to September 2016, there were 48,372 hospital admissions and 1071 (2.2%) in-hospital deaths. Deaths were preceded by a DNR order in 276 (25.8%) cases, and an admission to the ICU occurred in 814 (76%) cases. There was a mean of 36 ± 7 in-hospital deaths per month in the pre-implementation phase and 38 ± 6 in-hospital deaths per month in the post-intervention phase (p = 0.704).

Description of the demographic and clinical characteristics of patients who died in the hospital in the study period is depicted in table 1.

**Table 1:** Characteristics of the cohort, stratified by do-not-resuscitate status

Characteristics	Non-DNR (N = 795)	DNR (N = 276)	p value
Age (years)	69 (55 - 81)	64 (52.2 - 76)	0.01
Male sex	398 (50.1)	116 (42.0)	0.025
Non-elective (acute) admission	721 (90.7)	276 (100)	< 0.001
Primary admitting team			< 0.001
Surgical specialties	127 (16.0)	3 (1.1)	
Medical specialties	448 (56.4)	26 (9.4)	
Oncological specialties	220 (27.7)	247 (89.5)	
ICU admission during hospitalization	697 (87.7)	117 (42.4)	< 0.001
Setting of DNR order placement			NA
Ward	NA	199 (72.1)	
ICU	NA	63 (22.8)	
Intermediate-care unit	NA	14 (5.1)	
Death in the ICU	607 (76.4)	45 (16.3)	<0.001
Length of stay in the hospital (days)	13 (4 - 29)	12 (5.2 - 24.0)	0.592
Days from admission to DNR order	NA	5 (1.0 - 15.7)	NA
Days from DNR order to death	NA	4 (2-9)	NA

DNR - do-not-resuscitate; ICU - intensive care unit; NA - not applicable. Results expressed as the median (interquartile range) or number (%).

Patients with DNR orders were younger and more frequently female, had non-elective (acute) admissions, were more often admitted to oncological specialties and were less frequently admitted to the ICU in comparison to patients without DNR orders (Table 1).

Most DNR orders were placed in the wards, and patients with DNR orders were less likely to die in the ICU [OR (95%CI) = 0.06 (0.04 - 0.09), p < 0.001]. All patients with DNR orders who died in the ICU (45 patients, 71.4%) had their DNR orders placed in the ICU. However, 18 patients (28.6%) who had their DNR orders placed in the ICU were discharged and died in the wards.

Median (IQR) time from hospital admission to DNR order registration was 5 (1 - 16) days and median (IQR) time from DNR registration to death was 4 (2 - 9) days. Additionally, there was no difference in length of hospital stay for DNR and non-DNR patients (Table 1).

Characteristics of patients in the pre-implementation and post-implementation periods are described in table 2. Age and sex distribution were similar; however patients in the post-implementation period were more likely to be admitted to oncological specialties as the primary admitting team.

**Table 2:** Characteristics and outcomes of patients before (pre-implementation) and after (post-implementation) the implementation of the palliative care program

Characteristic	Pre-implementation (N = 613)	Post-implementation (N = 458)	p value
Age (years)	68 (54 - 81)	66.5 (53 - 79)	0.32
Male sex	287 (46.8)	227 (49.6)	0.374
Non-elective (acute) admission	576 (94)	421 (91.9)	0.192
Oncological specialties as primary team	251 (40.9)	216 (47.2)	0.042
ICU admission during hospitalization	469 (76.5)	345 (75.3)	0.654
DNR order	125 (20.4)	151 (33)	< 0.001
Setting of DNR order placement			0.931
Ward	90 (72)	109 (72.2)	
ICU	28 (22.4)	35 (23.2)	
Intermediate-care unit	7 (5.6)	7 (4.6)	
Death in the ICU	377 (61.5)	275 (60)	0.629
Length of stay in the hospital (days)	13 (5 - 29)	12 (5 - 27)	0.483
Days from admission to DNR order	5 (1 - 16)	6 (1 - 15)	0.921
Days from DNR order to death	3 (1.5 - 7)	4 (2 - 10)	0.157

ICU - intensive care unit; DNR - do-not-resuscitate. Results expressed as the median (interquartile range) or number (%).

After the implementation of the PC program, there was an increase in the proportion of in-hospital deaths with DNR order registration from 125 (20.4%) to 151 (33%) in the pre-implementation and post-implementation periods, respectively (p < 0.001).

**Figure 1:**
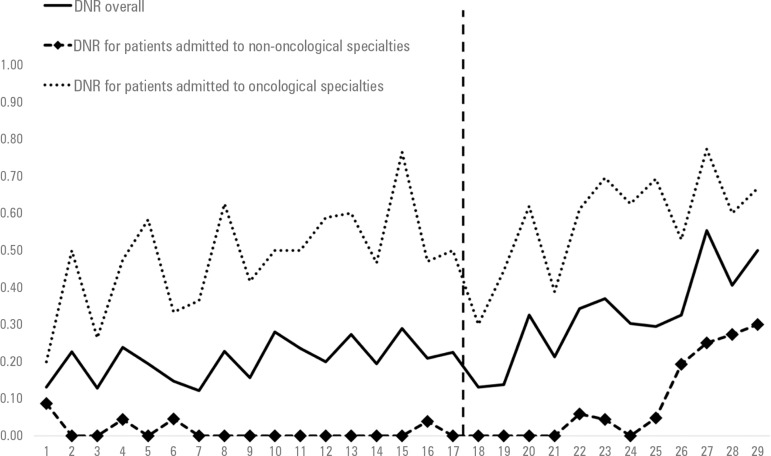
Trends in the proportion of do-not-resuscitate orders among in-hospital deaths before and after implementation of a palliative care program, overall (p < 0.001) and stratified by admission to oncological specialties (p = 0.149) and admission to non-oncological specialties (p < 0.001). DNR - do-not-resuscitate.

Mean ± SD proportion of in-hospital deaths with DNR orders was 0.20 ± 0.05 per month in the pre-implementation period and 0.32 ± 0.12 per month in the post-implementation period (p = 0.037). A stepped increase in DNR order registrations was seen after the implementation of the PC program in September 2015 (Figure 1), which was confirmed with interrupted time series analysis. Before the implementation of the PC program, the secular trend of increase in the proportion of in-hospital deaths with DNR orders was 0.5% per month (95%CI = 0.4 to 0.6), and after the implementation of the palliative care program, the trend increased to 2.9% per month (95%CI = 2.6 to 3.2), p < 0.001.

We performed analyses stratified for primary admitting teams. Patients admitted to oncological specialties were younger, with a median (IQR) age of 64 (52 - 75) years, whereas patients admitted to non-oncological specialties presented with a median age of 72 (55 - 83) years (p < 0.001). Patients admitted to oncological specialties were admitted to the ICU during hospitalization in 259 (55.5%) cases, and patients admitted to non-oncological specialties in 555 (91.9%) cases (p < 0.001). Admission to oncological specialties was associated with a lower chance of death in the ICU when compared to patients admitted to non-oncological specialties, with 178 (38.1%) and 474 (78.5%) cases, respectively.

Patients admitted to oncological specialties were more likely to have DNR orders when compared to non-oncological specialties, with 247 (52.9%) and 29 (4.8%) cases, respectively (p < 0.001). DNR orders were more likely to be placed in the wards for patients admitted to oncological specialties, with 191 (77.3%) cases, than in patients admitted to non-oncological specialties, with 8 (27.6%) cases (p < 0.001). DNR orders were also placed earlier for patients admitted to oncological specialties, with a median of 5 (1 - 15) days from admission *versus* 19 (6 - 31.5) days in patients admitted to non-oncological specialties (p < 0.001).

Of the 29 patients admitted to non-oncological specialties who had a DNR order placed, 19 had an acute diagnosis of sepsis, 5 were admitted for stroke, 3 for respiratory failure and 2 for other acute diagnoses. Of those 29 patients, 5 had a previous diagnosis of dementia, 5 were elderly frail patients, 4 presented with chronic obstructive pulmonary disease, 3 with cirrhosis, 2 with chronic kidney failure, 2 with heart failure, 1 did not have any other comorbidities and 7 had other diagnoses. Those patients were admitted to internal medicine (7; 24.1%), pneumology (5; 17.2%), neurology (5; 17.2%), general surgery (2; 6.9%), gastroenterology (2; 6.9%), hematology (2; 6.9%), nephrology (2; 6.9%), pediatrics (2; 6.9%), orthopedics (1; 3.4%) and cardiology (1; 3.4%).

The primary admitting team modified the effect of the PC program on DNR orders. Patients admitted to non-oncological specialties presented with a mean proportion of in-hospital deaths with DNR orders of 0.01 ± 0.03 per month in the pre-implementation period and 0.10 ± 0.12 per month in the post-implementation period (p = 0.009). On the other hand, there was no significant change for patients admitted to oncological specialties, who presented with a mean proportion of in-hospital deaths with DNR orders of 0.48 ± 0.14 per month in the pre-implementation period and 0.58 ± 0.14 per month in the post-implementation period (p = 0.07).

Interrupted time series analyses confirmed those results. For patients admitted to non-oncological specialties, the pre-implementation trend was a decrease of DNR orders of -0.4% per month (95%CI = -0.6 to - 0.2), and after the implementation, the trend was an increase of DNR orders of 2.8% per month (95%CI 2.4 to 3.2), p < 0.001. For patients admitted to oncological specialties, the pre-implementation trend was an increase of DNR orders of 1.4% per month (95%CI = 1.1 to 1.7), and the post-implementation trend was not significantly different, with an increase of 2.6% per month (95%CI = 2.1 to 3.1), p = 0.149.

Overall, ICU admission during hospitalization occurred in 469 (76.5%) patients in the pre-implementation period and in 345 (75.3%) patients in the post-implementation period (p = 0.654) (Table 2). There was also no change in the proportion of deaths in the ICU, which occurred in 377 (61.5%) patients in the pre-implementation period and in 275 (60%) patients in the post-implementation period (p = 0.629).

The mean ± SD of in-hospital deaths with ICU admission during the hospitalization was 0.76 ± 0.04 per month in the pre-implementation period and did not change in the post-implementation period, in which the proportion was 0.76 ± 0.07 per month (p = 0.778).

**Figure 2:**
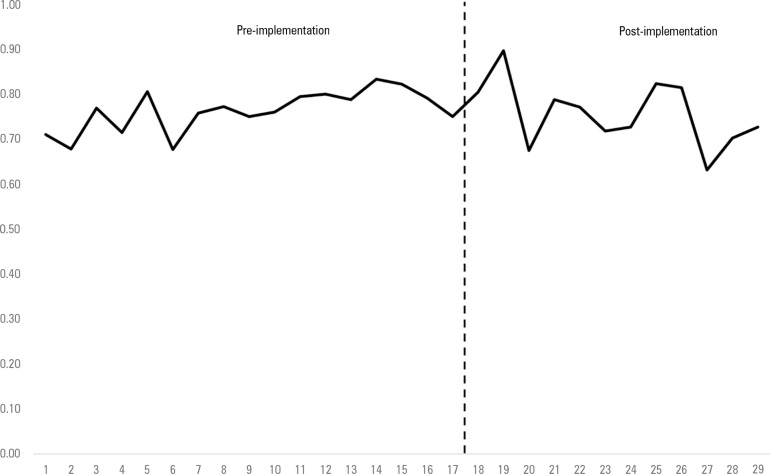
Trend in the proportion of intensive care unit utilization among in-hospital deaths before and after implementation of a palliative care program, p = 0.001 for a change in slope.

Interrupted time series analyses, however, demonstrated a change in the slope of the trend of ICU admission. In the pre-implementation phase, there was an increase of 0.6% per month (95%CI = 0.5 to 0.7) of ICU admissions among in-hospital deaths (Figure 2). After the implementation of the PC program, the trend was a decrease in ICU admissions among in-hospital deaths of -0.9% per month (95%CI = -1.2 to -0.6; p = 0.001).

## DISCUSSION

In the present study, the implementation of a PC program was associated with an increased trend in prescription of DNR orders, especially in patients admitted to non-oncological specialties, and a decreased trend in ICU utilization during terminal hospitalizations. However, there was a high rate of intensive care unit utilization in patients dying in the hospital, even though patients with DNR orders were less likely to die in the ICU.

Palliative care has been suggested to improve communication, definitions of goals-of-care and to alleviate distressing symptoms in terminal phases of diseases.^(1)^ Because hospitals are a major site for end-of-life care,^(2-4)^ interventions to improve PC during terminal hospitalizations have been implemented and analyzed in the literature.^(13-15)^

Our results are aligned with the literature on the impact of PC interventions, in which PC has been associated with modifications in goals-of-care definitions and resource utilization.^(28,29)^ For example, in a systematic review, it was demonstrated that PC interventions are associated with a reduction of 37% in the chance of ICU admission.^(14)^ Nevertheless, in the present study, the rate of ICU admission during terminal hospitalization and death in the ICU were high, at 76% and 61%, respectively. Even among DNR patients, ICU admission and death in the ICU approached 42% and 16%, respectively. Although high, our results are not very different from previous studies, which demonstrate that almost one third of patients with serious illnesses die in the ICU^(30)^ or with ventilator support.^(20)^

Our data demonstrated that the implementation of a PC program increased DNR orders in patients admitted to non-oncological specialties but had no effect on patients admitted to oncological specialties. It has been shown before that the diagnosis of cancer influences end-of-life care, with cancer patients receiving, generally, less aggressive care and better quality of end-of-life care.^(5,30,31)^ It has been hypothesized that some of this difference may be due to misunderstandings about the trajectory of the disease or lack of familiarity with PC by the providers.^(31)^ It is possible that the implementation of the program had a greater effect in this more vulnerable population of non-cancer patients, as cancer patients may have already been receiving more appropriate end-of-life care, even though this correlation should be carefully examined.

Our study was one of the few to analyze ICU utilization during terminal hospitalizations and to analyze a PC intervention in a developing country.^(32,33)^ Moreover, to our knowledge, this study is the first to demonstrate that a hospital PC program had an impact on outcomes in Brazil, even though it is a country in which it has been shown that quality of end-of-life care may be poor and PC of limited access.^(34)^ Additionally, it has been suggested that there are differences between Brazil and other countries in things people seem to give importance to at the end of life,^(35)^ such as the setting of death,^(36)^ with Brazilians valuing more "living as long as possible" and being more likely to die in the hospital.

Another strength of this study is that we utilized interrupted time series analyses to evaluate the trends of DNR orders and ICU admissions. Interrupted time series analysis is a robust method, which has been considered the "next best approach for dealing with interventions when randomization is not possible or clinical data is not available".^(27)^ This method of analysis allows for the investigation of potential biases that are common in implementation studies, such as the secular trend bias, in which the outcome may be increasing or decreasing with time, irrespective of the intervention implemented.^(37)^

However, our study has some limitations. This study was performed in only one center, so its generalizability may be reduced. Nevertheless, we have analyzed a large number of in-hospital deaths for 29 months, and our results may be generalized to other similar settings. This study was also retrospective and based on electronic health record analyses, which may be subject to biases. Another limitation is that we were not able to retrieve more specific information on the quality of end-of-life care of the patients enrolled.

## CONCLUSION

The implementation of a palliative care program was associated with a trend of increased registration of do-not-resuscitate orders and a decrease of intensive care unit utilization during terminal hospitalizations. However, the increased registration of do-not-resuscitate orders after the implementation was seen in patients admitted to non-oncological specialties but not in patients admitted to oncological specialties.

### Authors' contributions

JGR Ramos and FC Tourinho contributed to the design, acquisition, analysis and interpretation of data and the drafting and revising of the manuscript. P Borrione, PBP Batista and AV Mendes contributed to the analysis and interpretation of data and the drafting and revising of the manuscript. P Azi, V Costa, T Andrade and Z Reis contributed to the conception of the study and critical revision of the manuscript. All authors have approved the final version of the manuscript.

## References

[1] Blinderman CD, Billings JA (2015). Comfort care for patients dying in the hospital. N Engl J Med.

[2] Hall MJ, Levant S, DeFrances CJ (2013). Trends in inpatient hospital deaths: National Hospital Discharge
Survey, 2000-2010. NCHS Data Brief.

[3] Angus DC, Barnato AE, Linde-Zwirble WT, Weissfeld LA, Watson RS, Rickert T, Rubenfeld GD, Robert Wood Johnson Foundation ICU End-Of-Life Peer Group (2004). Use of intensive care at the end of life in the United States: an
epidemiologic study. Crit Care Med.

[4] Weitzen S, Teno JM, Fennell M, Mor V (2003). Factors associated with site of death: a national study of where
people die. Med Care.

[5] Lyngaa T, Christiansen CF, Nielsen H, Neergaard MA, Jensen AB, Laut KG (2015). Intensive care at the end of life in patients dying due to
non-cancer chronic diseases versus cancer: a nationwide study in
Denmark. Crit Care.

[6] (1995). A controlled trial to improve care for seriously ill hospitalized
patients. The study to understand prognoses and preferences for outcomes and
risks of treatments (SUPPORT). The SUPPORT Principal
Investigators. JAMA.

[7] Schneiderman LJ, Gilmer T, Teetzel HD, Dugan DO, Blustein J, Cranford R (2003). Effect of ethics consultations on nonbeneficial life-sustaining
treatments in the intensive care setting: a randomized controlled
trial. JAMA.

[8] Kon AA, Shepard EK, Sederstrom NO, Swoboda SM, Marshall MF, Birriel B (2016). Defining futile and potentially inappropriate interventions: a
policy statement from the Society of Critical Care Medicine Ethics
Committee. Crit Care Med.

[9] Adhikari NK, Fowler RA, Bhagwanjee S, Rubenfeld GD (2010). Critical care and the global burden of critical illness in
adults. Lancet.

[10] Halpern NA, Pastores SM (2010). Critical care medicine in the United States 2000-2005: an
analysis of bed numbers, occupancy rates, payer mix, and
costs. Crit Care Med.

[11] Sprung CL, Baras M, Iapichino G, Kesecioglu J, Lippert A, Hargreaves C (2012). The Eldicus prospective, observational study of triage decision
making in European intensive care units: part I--European Intensive Care
Admission Triage Scores. Crit Care Med.

[12] Quill CM, Ratcliffe SJ, Harhay MO, Halpern SD (2014). Variation in decisions to forgo life-sustaining therapies in US
ICUs. Chest.

[13] Martins BD, Oliveira RA, Cataneo AJ (2017). Palliative care for terminally ill patients in the intensive care
unit: Systematic review and metaanalysis. Palliat Support Care.

[14] Khandelwal N, Kross EK, Engelberg RA, Coe NB, Long AC, Curtis JR (2015). Estimating the effect of palliative care interventions and
advance care planning on ICU utilization: a systematic
review. Crit Care Med.

[15] Khandelwal N, Benkeser DC, Coe NB, Curtis JR (2016). Potential influence of advance care planning and palliative care
consultation on ICU costs for patients with chronic and serious
illness. Crit Care Med.

[16] Aslakson RA, Curtis JR, Nelson JE (2014). The changing role of palliative care in the ICU. Crit Care Med.

[17] De Lima L, Bruera E (2000). The Pan American Health Organization: its structure and role in
the development of a palliative care program for Latin America and the
Caribbean. J Pain Symptom Manage.

[18] Gelband H, Sankaranarayanan R, Gauvreau CL, Horton S, Anderson BO, Bray F, Cleary J, Dare AJ, Denny L, Gospodarowicz MK, Gupta S, Howard SC, Jaffray DA, Knaul F, Levin C, Rabeneck L, Rajaraman P, Sullivan T, Trimble EL, Jha P, Disease Control Priorities-3 CancerAuthor Group (2016). Costs, affordability, and feasibility of an essential package of
cancer control interventions in low-income and middle-income countries: key
messages from Disease Control Priorities, 3rd edition. Lancet.

[19] Harding R, Gwyther L, Mwangi-Powell F, Powell RA, Dinat N (2010). How can we improve palliative care patient outcomes in low- and
middle-income countries? Successful outcomes research in sub-Saharan
Africa. J Pain Symptom Manage.

[20] Cheng MT, Hsih FY, Tsai CL, Tsai HB, Tsai DF, Fang CC (2016). Increased rate of DNR status in hospitalized end-of-life patients
in Taiwan. Intensive Care Med.

[21] Souza PN, Miranda EJ, Cruz R, Forte DN (2016). Palliative care for patients with HIV/AIDS admitted to intensive
care units. Rev Bras Ter Intensiva.

[22] Mazutti SR, Nascimento AF, Fumis RR (2016). Limitation to Advanced Life Support in patients admitted to
intensive care unit with integrated palliative care. Rev Bras Ter Intensiva.

[23] De Simone GG (2003). Palliative care in Argentina: perspectives from a country in
crisis. J Pain Palliat Care Pharmacother.

[24] Maharaj S, Harding R (2016). The needs, models of care, interventions and outcomes of
palliative care in the Caribbean: a systematic review of the
evidence. BMC Palliat Care.

[25] Nervi F, Guerrero M, Reyes MM, Nervi B, Cura A, Chávez M (2003). Symptom control and palliative care in Chile. J Pain Palliat Care Pharmacother.

[26] Cochrane Effective Practice and Organisation of Care (EPOC) EPOC Resources for review authors. Interrupted time series (ITS)
analyses.

[27] Kontopantelis E, Doran T, Springate DA, Buchan I, Reeves D (2015). Regression based quasi-experimental approach when randomisation
is not an option: interrupted time series analysis. BMJ.

[28] Gade G, Venohr I, Conner D, McGrady K, Beane J, Richardson RH (2008). Impact of an inpatient palliative care team: a randomized control
trial. J Palliat Med.

[29] Picker D, Dans M, Heard K, Bailey T, Chen Y, Lu C (2017). A Randomized Trial of Palliative Care Discussions Linked to an
Automated Early Warning System Alert. Crit Care Med.

[30] Wachterman MW, Pilver C, Smith D, Ersek M, Lipsitz SR, Keating NL (2016). Quality of end-of-life care provided to patients with different
serious illnesses. JAMA Intern Med.

[31] Koff G, Vaid U, Len E, Crawford A, Oxman DA (2017). Differences in utilization of life support and end-of-life care
for medical ICU patients with versus without cancer. Crit Care Med.

[32] Paiva CE, Faria CB, Nascimento MS, Dos Santos R, Scapulatempo HH, Costa E (2012). Effectiveness of a palliative care outpatient programme in
improving cancer-related symptoms among ambulatory Brazilian
patients. Eur J Cancer Care (Engl).

[33] Soares LG, Japiassu AM, Gomes LC, Pereira R (2018). Post-acute care facility as a discharge destination for patients
in need of palliative care in Brazil. Am J Hosp Palliat Care.

[34] Economist Inteligence Unit (EIU) (2015). The 2015 Quality of Death Index. Ranking palliative care across the
world. The Economist.

[35] (2017). What people want most in their final months. The Economist.

[36] (2017). A better way to care for the dying. The Economist.

[37] Ramsay CR, Matowe L, Grilli R, Grimshaw JM, Thomas RE (2003). Interrupted time series designs in health technology assessment:
lessons from two systematic reviews of behavior change
strategies. Int J Technol Assess Health Care.

